# Supplementary material for: It’s not who you know, but who you are: Explaining income gaps of stigmatized-caste business owners in India
Source: PLoS One. 2024 Aug 7;19(8):e0307660. doi: 10.1371/journal.pone.0307660 (PMC11305585; doi:10.1371/journal.pone.0307660)
Supplement: S1 File — (DOCX) [file pone.0307660.s002.docx]

**SUPPLEMENTARY INFORMATION**

***Control Variable(s)***

We control for the following control variables.

*Untouchability experienced by household and caste:* The experience of untouchability is distinct and direct, unlike institutional stigma that operates in invisible fashion and may not be noticed or felt by stigmatized individuals. Therefore, controlling for experiences of untouchability helps us ascertain if stigma indeed does disadvantage business owners at an institutional level, as we predict it will. The IHDS survey asks Dalit households if members of their households have experienced untouchability in the past five years. We use this information to construct the variable *Untouchability experienced by household*. Further, we also calculate the average untouchability experienced by all households (i.e., both business owning and non-business owning) of a given caste, as reported across all respondents in the IHDS survey, and use this variable to control for the experienced untouchability of the caste.

*Socioeconomic disadvantage:* Besides Dalits, other castes/tribes/groups that face socioeconomic disadvantage are Other Backward Castes (OBCs), Adivasis (Scheduled Tribes), and Muslim households. To reliably estimate the effect of institutional stigma (and rule out the effects of socioeconomic disadvantage in general) on business income, we add a dummy variable to control for households that belong to OBC, Adivasi, or Muslim communities.

*Location characteristics:* To control for the effect of locational characteristics on business income and inequalities [1], we control for three variables: the type of location (urban vs. rural), average income in the location, and the fraction of Dalits in the location, as codified in our data source. First, we control for *Urban* nature (with a dummy coded 1 if the household is in an urban region of the district) because urban economies are usually more prosperous, which is also likely to have implications for businesses operating in urban location. Additionally, this dummy variable also allows us to account for further locational characteristics within each given district. Second, we control for *Average income – Location*, that measures the average per capita income (log transformed) of all households (business owners and non-business owners) in the neighborhood/village of a household. Third, a large percentage of Dalits in a locality might reduce the economic potential of the area. Therefore, we control for *Fraction of Dalits in location,* measured as the number of Dalit households sampled in that village or neighborhood divided by the total number of households in that location.

*Household characteristics*. We control for the effect of household characteristics on business income with four variables: household size, number of businesses owned by the household, land ownership, and tenure of residence. First, we control for *Household size*, captured as the number of people in household. Second, we control for *Number of businesses in household,* captured as the number of non-farm businesses owned by the members of household. Third, we control for *Land*, captured in whether the household has any owned or cultivated land (dummy variable coded as 1, if yes). Given the largely rural nature of India, land is an important asset that households can use as a collateral to raise capital. Alternatively, land can also tie households to their rural and semi-urban backgrounds, reducing their propensity to migrate to urban centres and/or pursue entrepreneurship. Finally, we control for *Tenure of residence*, measured as the number of years the household lived in the district, as this can influence their business performance through multiple mechanisms, such as reputation, familiarity with business norms, and so on.

***Fixed Effects***

In addition to the control variables described above, we account for *Father’s Occupation*, *District*, and *Industry* using a set of indicators or dummy variables, i.e., fixed effects. First, in India, as in some other patriarchal societies, father’s occupation can act as a proxy for the socio-economic class a person was raised into. While biographical accounts are ripe with many instances where entrepreneurs from marginalized backgrounds “defy the odds” and find success without a supportive family background, a broader empirical literature shows family history of occupation has a bearing on an individual’s outcomes [2]. The IHDS codifies occupation of household head’s father (or the household head’s husband if the head is a woman) into 116 categories. Using father’s occupation to account for intergenerational class is common in studies of inequality and social mobility [e.g., 2, 3]. Second, over and above the controls for location, the district fixed effects can account for the socio-economic, political, and cultural differences across regions that may influence business income, and more pertinently the relationship between stigma and business income. The IHDS data come from 373 districts, and we control for the possible impact of a household’s geographic location by adding district fixed effect. Finally, to account for the industry-level variation in business income, as well as the differential effect that institutional stigma may have on business income in different industries, we added industry fixed effects (i.e., 67 classified industries, as per IHDS data).

***Summary Statistics***

We present in Table A1 the means, standard deviations, and correlations for the variables in our regression models. The IHDS variables we use are fact-based (main variables are business income, demographic group, highest adult education, and acquaintances to members of specific professions) [4], and collected in 13 different local languages to minimize item ambiguity [5]. The strong correlations between our variables of interest (i.e., 0.6 and above) are only between variables that are drawn from the same conceptual space, such as social capital and bonding and bridging social capital, and untouchability experienced at the household and caste levels. Hence, our variables are within reasonable limits of correlation, reducing the likelihood of common method bias (CMB) [6, 7]. On conducting the Harman single factor test for all the variables listed in Table 1, we find that 5 factors have eigenvalues greater than 1, which together account for 62.3% of the variance. The factor with the largest eigenvalue accounted for only 21.4% of the variance. These figures also suggest that a substantial amount of CMB is unlikely [4]. As the risk of CMB is reduced by specifying complex relationships that are unlikely to be a part of the respondents’ mental maps [e.g., 5], we use interaction effects (to test H2, H3, and H4), reducing the possibility that our results are an artifact of the respondents’ mental maps.

The correlations that are above 0.60 are between *Bonding* and *Bridging social capital*, on the one hand, and *Social capital*, on the other; and that between the untouchability that is experienced in one’s own household and the average level of untouchability experienced in one’s community. In this latter case, some correlation between those two variables is expected, and our results regarding hypothesized relationships remain the same if we remove either of these variables across our models (we prefer to keep both in the results we present since this allows us to control more carefully for possible confounds and the correlation is not problematically large). In the case of the former correlations, in our models we enter either *Social capital* on its own or its constituents, i.e., *Bridging* and *Bonding social capital*, but not all three together (since the latter two make up overall *Social capital*). In terms of the variance inflation factors (VIFs), the average VIF is below 10 in all of our models. More specifically to our hypothesized effects, the VIF for any of the social capital variables, human capital, or institutional stigma, are not above 2.5 in any of our models.

The negative correlations between *Institutional stigma* with the three social capital variables and with human capital are consistent with our expectation, and with research that has studied disadvantaged communities in other settings. Comparisons between the groups, based on *t*-tests of means, confirms this pattern, that groups that have *Institutional stigma* have lower levels of social and human capital (*p* < .001). Our predictions are about the returns to a given level of these forms of capital, which we test with interaction variables. The variables for social capital and human capital in the models control for differences in levels (or “holds them constant”), when we are investigating the business income implications of possible differences in returns with the interaction variables.

1. Chetty, R., Hendren, N., & Katz, L.F. 2016. The effects of exposure to better neighborhoods on children: New evidence from the Moving to Opportunity experiment. ***American Economic Review***, *106*(4): 855-902.
2. Long, J., & Ferrie, J. 2013. Intergenerational occupational mobility in Great Britain and the United States since 1850. ***American Economic Review***, *103*(4): 1109-37.
3. Kish-Gephart, J.J. and Campbell, J.T. 2015. You don’t forget your roots: The influence of CEO social class background on strategic risk taking. ***Academy of Management Journal***, *58*(6): 1614-1636.
4. Podsakoff, P.M., MacKenzie, S.B., Lee, J.Y., & Podsakoff, N.P. 2003. Common method biases in behavioral research: a critical review of the literature and recommended remedies. ***Journal of Applied Psychology***, *88*(5): 879.
5. Chang, S.J., Van Witteloostuijn, A., & Eden, L. 2010. From the editors: Common method variance in international business research. ***Journal of International Business Studies***. *41*: 178-184
6. Bagozzi, R.P., Yi, Y., & Phillips, L.W. 1991. Assessing construct validity in organizational research. ***Administrative Science Quarterly***, 36(3): 421-458.
7. Tehseen, S., Ramayah, T., & Sajilan, S. 2017. Testing and controlling for common method variance: A review of available methods. ***Journal of Management Sciences***, *4*(2): 142-168.

**Table A1: Means, Standard Deviations, and Correlations**

|  | **Variables** | **Mean** | **Std. dev** | **1** | **2** | **3** | **4** | **5** | **6** | **7** | **8** | **9** | **10** | **11** | **12** | **13** | **14** | **15** |
| --- | --- | --- | --- | --- | --- | --- | --- | --- | --- | --- | --- | --- | --- | --- | --- | --- | --- | --- |
| 1 | Business income (log) | 10.773 | 1.392 |  |  |  |  |  |  |  |  |  |  |  |  |  |  |  |
| 2 | Institutional Stigma (Dalit) | .139 | .346 | -.103 |  |  |  |  |  |  |  |  |  |  |  |  |  |  |
| 3 | Social capital | 4.538 | 4.232 | .221 | -.084 |  |  |  |  |  |  |  |  |  |  |  |  |  |
| 4 | Bonding social capital | 1.756 | 2.188 | .207 | -.070 | .844 |  |  |  |  |  |  |  |  |  |  |  |  |
| 5 | Bridging social capital | 2.781 | 2.659 | .182 | -.076 | .897 | .520 |  |  |  |  |  |  |  |  |  |  |  |
| 6 | Human capital | 9.904 | 4.601 | .292 | -.120 | .354 | .318 | .311 |  |  |  |  |  |  |  |  |  |  |
| 7 | Untouchability experience - Household | .025 | .155 | -.075 | .394 | -.068 | -.064 | -.064 | -.101 |  |  |  |  |  |  |  |  |  |
| 8 | Untouchability experience - Caste (avg.) | .028 | .105 | -.084 | .554 | -.081 | -.074 | -.073 | -.109 | .629 |  |  |  |  |  |  |  |  |
| 9 | Socioeconomic disadvantage | .708 | .455 | -.178 | .259 | -.187 | -.188 | -.158 | -.307 | .102 | .152 |  |  |  |  |  |  |  |
| 10 | Tenure of residence | 73.725 | 27.635 | -.125 | .005 | -.037 | -.041 | -.027 | -.120 | .031 | .024 | .108 |  |  |  |  |  |  |
| 11 | Urban location | .511 | .500 | .267 | -.034 | .084 | .090 | .063 | .235 | -.041 | -.045 | -.140 | -.368 |  |  |  |  |  |
| 12 | Fraction of Dalits in location | .188 | .217 | -.104 | .512 | -.090 | -.088 | -.090 | -.107 | .199 | .279 | .120 | .024 | -.142 |  |  |  |  |
| 13 | Average income - Location | 9.756 | .694 | .361 | -.035 | .190 | .187 | .178 | .326 | -.077 | -.070 | -.260 | -.260 | .466 | -.099 |  |  |  |
| 14 | Land | .341 | .474 | -.129 | -.060 | .034 | .030 | .029 | -.008 | -.021 | -.029 | .011 | .253 | -.501 | .047 | -.289 |  |  |
| 15 | Number of businesses in household | 1.116 | .351 | .189 | -.019 | .082 | .064 | .063 | .085 | -.020 | -.033 | -.013 | -.004 | .043 | .0002 | .023 | -.001 |  |
| 16 | Household size | 5.424 | 2.554 | .116 | -.013 | .060 | .045 | .045 | .123 | -.012 | -.012 | .063 | .089 | -.035 | .005 | -.092 | .132 | .173 |
| *Notes.* N = 8,506. Correlations stronger than \|.021\| are significant at *p* *<* .05 | | | | | | | | | | | | | | | | | | |
