# Supplementary material for: It’s not who you know, but who you are: Explaining income gaps of stigmatized-caste business owners in India
Source: PLoS One. 2024 Aug 7;19(8):e0307660. doi: 10.1371/journal.pone.0307660 (PMC11305585; doi:10.1371/journal.pone.0307660)
Supplement: Attachment — Submitted filename: Response to Reviewers.docx [file pone.0307660.s003.docx]

**Response to the comments**

We thank the editor and the reviewer for reviewing our manuscript. The comments helped us improve the manuscript and “*reflect more on the meaning of diffused prejudice and stigmatized identity*.” We have addressed the comments below and have made the changes in the main draft accordingly. We kindly request you to go through our point-by-point responses to the comments.

Our replies to the reviewers’ comments are shown in red. Our revised text is shown in blue and also highlighted in the main draft.

Reviewer 1: “*Mention introduction giving the significance and objective of your study. The first paragraph must be in your own words without citations*.”

Thank you for this suggestion. We have added a first paragraph (reproduced below) in our own words and without citations, which clarifies the significance and objective of the study.

The World Inequality Report 2022 reported that the top 10% of earners took home 52% of the global income, while the bottom 50% earned just 8% of the total income. Understanding the antecedents of such stark income inequalities is a major challenge of our times. Systematic and granular studies documenting inequality generating processes are important to grasp this complex issue. This study addresses the issue of income disadvantages faced by business owners from stigmatized castes in India. Despite the policy focus on reducing income disparities through human and social capital, it remains unclear whether these strategies are equally effective for marginalized groups in societies with systemic inequalities. This study aims to fill this gap by analyzing data from a nationwide survey to quantify the income disparity faced by Dalit business owners compared to other disadvantaged but non-stigmatized groups.

Reviewer 1: “*Literature review and methodology section is missing. Give a structured LR and explain the research methodology clearly. Mention hypothesis in the methodology section*.”

Thank you for these very helpful suggestions. We have added a new section “Literature Review” with five paragraphs of literature review giving a background on the inequality literature, stigma, and the caste system. We have also added a separate “Methodology” section that first includes the hypotheses, followed by an exposition of the method. These changes have been tracked in the revised document.

Reviewer 1: “*Future scope of research is missing*.”

Thank you for this suggestion. We have added a paragraph on the future scope of work as the second paragraph of our Discussion section.

Our findings should be interpreted in the context of their limitations. The measure of social capital that is available in this survey captures the presence of ties to different groups and professions, but not their number or strength. Therefore, it is a coarse indicator of social capital, but one that presents a reasonable trade-off, given the coverage provided in the survey and also in light of all the other information asked about in this survey (much of which we use to improve the specification of our model and control for possible confounding variables). We also note that the cross-sectional data limits the extent to which we can make causal inferences, although we control for a number of household, neighborhood, industry, and regional characteristics, and even though in this case the institutional stigma indicator we use is *highly* unlikely to change as a result of changes in the outcome we study. Notwithstanding these limitations, our study makes contributions to research on economic inequality, as well as to stigma research, and has implications for management practice and policy.

Reviewer 2: “*One, Social Capital (SC) as a framework may not be appropriate in a caste-infested country. Even in America, SC primarily has been a factor among one social group, the WASPs. I maybe wrong but I feel Blacks cannot join a tennis club or a neighborhood volunteer group or a church and later use it as a vehicle for career advancement. So, in a homogeneous group bridging SC is easy. Caste is heterogeneous. SC is a class concept*.”

Thank you for this very useful perspective. We acknowledge this now in our manuscript, and to further accentuate your points we added the following text to highlight some mechanisms which render social capital ineffective for stigmatized communities.

In the context of India, while non-stigmatized groups may benefit from their social capital, Dalit business owners will likely receive lower returns for their social capital due to frequent rejections, micro-aggressions, and the burden of managing their stigmatized identity, which hinders their ability to secure valuable business opportunities (Jones & King, 2014; Meuris & Leana, 2018).

Reviewer 2: “*Two, father's occupation is no predictor of mobility. Defying the Odds by Devesh Kapur et al chronicles how first-generation, (mostly) illiterate and landless Dalits succeeded as entrepreneurs - without hiding their identity*.”

Thank you for this comment, which allowed us to revisit the book Kapur, Babu and Prasad (2014). We draw on the discussion in this book, which highlighted that many successful entrepreneurs defy the odds as first generation entrepreneurs. We also add the caveat that empirical literature (e.g., Long and Ferrie, 2013) shows that success of individuals often relies heavily on their family’s occupational background.

While biographical accounts are ripe with many instances where entrepreneurs from marginalized backgrounds “defy the odds” (Kapur, Babu, and Prasad, 2014) and find success without a supportive family background, a broader empirical literature shows family history of occupation has a bearing on an individual’s outcomes (Long & Ferrie, 2013).

Reviewer 2: “*Three, having land (mostly in small pieces) hinders mobility and entrepreneurship as it ties them down to the same village*.”

Thank you. This could be an alternate mechanism through which ownership of land hinders joining entrepreneurship in high opportunity urban areas. We now acknowledge this possible mechanism.

Alternatively, land can also tie households to their rural and semi-urban backgrounds, reducing their propensity to migrate and pursue entrepreneurship.

Reviewer 2: “*Four, since entrepreneurship among Dalits is a new phenomenon since late 1990s, you may have compared first generation Dalit entrepreneurs with entrenched groups' who have been in businesses for generations, even if they are disadvantaged social groups*.”

Thank you for this very helpful critique. To address this issue we had run various matching algorithms as robustness tests. We directly address your concerns now with the following text, which now provides a clearer rationale behind conducting the robustness tests.

There may be genuine concerns whether comparing the entire samples of Dalit business owners to non-Dalit business owners in an OLS model, regardless of the battery of controls, is an apples-to-apples comparison. Hence, we also try to compare Dalit business owners to business owners that exhibit closely matching characteristics.

Reviewer 2: “*Five, some qualitative stuff adds luster to any work based on numbers*.”

Thank you for this suggestion. We agree that a qualitative insight would add depth to the paper. Following your suggestion we add a quote from Kapur, Babu, and Prasad (2014) in the methodology section which best highlights our paper’s central arguments.

Biographical accounts have highlighted the importance of social and human capital in the rise of business owners from Dalit communities. For example, Kapur, Babu and Prasad (2014), write in their biographic accounts of successful Dalit entrepreneurs emphasizing the role of both an entrepreneur’s immediate social capital and human capital;

“*As one reads the stories in this book, some commonalities emerge in the routes to success for these Dalits. Often, their success was nurtured by one or both parents (and especially the mother) who saw the benefit of providing their child with an education. They ensured funds for the child's education by taking on extra backbreaking work that provided the ready cash, sometimes choosing between their children when finances were severely strained and allowed the education of only one child. Often the siblings would also make sacrifices by taking less so that the pooled resources would be above a minimum threshold. This allowed a chosen child to access opportunities that would create a pathway to success for one member, who in time would hopefully 'pull up' the entire family.*

*A common thread among these stories is the jugaad employed by these budding entrepreneurs to find start-up funds for their new ventures. These funds are often provided by family and friends-the wife selling her jewellery, the mother relinquishing her savings, and other family members and well-wishers chipping in. Without this initial capital, it is unlikely that these fledgling entrepreneurs would have made a success of their ventures…The near absence of linkages with a financial system dominated by public sector banks, supposedly enjoined to lend for ‘priority sectors’, matters even more since Dalit entrepreneurs lack access to the sorts of social networks that are now recognized as critical for success in any walk of life, especially entrepreneurship.”*
